# *Triatoma dimidiata* Infestation in Chagas Disease Endemic Regions of Guatemala: Comparison of Random and Targeted Cross-Sectional Surveys

**DOI:** 10.1371/journal.pntd.0001035

**Published:** 2011-04-12

**Authors:** Raymond J. King, Celia Cordon-Rosales, Jonathan Cox, Clive R. Davies, Uriel D. Kitron

**Affiliations:** 1 Department of Infectious and Tropical Diseases, London School of Hygiene and Tropical Medicine, London, United Kingdom; 2 Center for Health Studies, Centers for Disease Control and Prevention for Central America and Panama (CDC-CAP), Universidad del Valle de Guatemala, Guatemala City, Guatemala; 3 Department of Environmental Studies, Math and Science Center, Emory University, Atlanta, Georgia, United States of America; Universidad de Buenos Aires, Argentina

## Abstract

**Background:**

Guatemala is presently engaged in the Central America Initiative to interrupt Chagas disease transmission by reducing intradomiciliary prevalence of *Triatoma dimidiata*, using targeted cross-sectional surveys to direct control measures to villages exceeding the 5% control threshold. The use of targeted surveys to guide disease control programs has not been evaluated. Here, we compare the findings from the targeted surveys to concurrent random cross-sectional surveys in two primary foci of Chagas disease transmission in central and southeastern Guatemala.

**Methodology/Principal Findings:**

Survey prevalences of *T. dimidiata* intradomiciliary infestation by village and region were compared. Univariate logistic regression was used to assess the use of risk factors to target surveys and to evaluate indicators associated with village level intradomiciliary prevalences >5% by survey and region. Multivariate logistic regression models were developed to assess the ability of random and targeted surveys to target villages with intradomiciliary prevalence exceeding the control threshold within each region. Regional prevalences did not vary by survey; however, village prevalences were significantly greater in random surveys in central (13.0% versus 8.7%) and southeastern (22.7% versus 6.9%) Guatemala. The number of significant risk factors detected did not vary by survey in central Guatemala but differed considerably in the southeast with a greater number of significant risk factors in the random survey (e.g. land surface temperature, relative humidity, cropland, grassland, tile flooring, and stick and mud and palm and straw walls). Differences in the direction of risk factor associations were observed between regions in both survey types. The overall discriminative capacity was significantly greater in the random surveys in central and southeastern Guatemala, with an area under the receiver-operator curve (AUC) of 0.84 in the random surveys and approximately 0.64 in the targeted surveys in both regions. Sensitivity did not differ between surveys, but the positive predictive value was significantly greater in the random surveys.

**Conclusions/Significance:**

Surprisingly, targeted surveys were not more effective at determining *T. dimidiata* prevalence or at directing control to high risk villages in comparison to random surveys. We recommend that random surveys should be selected over targeted surveys whenever possible, particularly when the focus is on directing disease control and elimination and when risk factor association has not been evaluated for all regions under investigation.

## Author Summary

Chagas disease is a vector-borne parasitic zoonosis endemic throughout South and Central America and Mexico. Guatemala is engaged in the Central America Initiative to interrupt Chagas disease transmission. A major strategy is the reduction of *Triatoma dimidiata* domiciliary infestations through indoor application of residual insecticides. Successful control of *T. dimidiata* will depend on accurate identification of areas at greatest risk for infestation. Initial efforts focused primarily on targeted surveys of presumed risk factors and suspected infestation to define intervention areas. This policy has not been evaluated and might not maximize the effectiveness of limited resources if high prevalence villages are missed or low prevalence villages are visited unnecessarily. We compare findings from the targeted surveys to concurrent random surveys in two primary foci of Chagas disease transmission in Guatemala to evaluate the performance of the targeted surveys. Our results indicate that random surveys performed better than targeted surveys and should be considered over targeted surveys when reliability of risk factors has not been evaluated, identify useful environmental factors to predict infestation, and indicate that infestation risk varies locally. These findings are useful for decision-makers at national Chagas Disease control programs in Central America, institutions supporting development efforts, and funding agencies.

## Introduction

In Guatemala, nearly 4 million individuals are projected to be at risk for infection with *Trypanosoma cruzi*, the causative agent of Chagas disease, with approximately 30,000 new cases a year and a prevalence of 730,000 [1], [2]. The estimated prevalence and annual incidence is more than double any other country in Central America and is substantially greater than that observed in Mexico [1], [2]. Based on the results of the national survey of triatomine populations conducted from 1995–8, the principal focus of transmission is considered to be in the southeastern and central departments of the country where the prevalence of triatomine vectors [3], the estimated human population at risk for *Trypanosoma cruzi* infection [3], and the infection rate of triatomine vectors with *T. cruzi* [4] is greatest[1]. This is also the region where the vector *Triatoma dimidiata* (Latreille 1811) is most abundant [3], [4], [5].

The Guatemalan National Ministry of Health (GNMH) is engaged in the Central America Initiative to interrupt Chagas Disease transmission (IPCA) [6], [7], [8], and Guatemala is the country with the most progress to date [9]. All available information indicates that *Rhodnius prolixus* has been eliminated (GNMH communication) and populations of the indigenous *T. dimidiata* have been reduced in the domestic environment three to nine fold [10], [11]. However, since *T. dimidiata* is a native species also occurring in the peridomestic and sylvatic environments, elimination is virtually impossible [2], [12], [13], [14]. Therefore, the goal is to reduce and maintain *T. dimidiata* village level intradomiciliary prevalence and colonization (nymphal intradomiciliary prevalence) below 5% [1], [2], [6], [7], [8], [11], [15].

Vector control relies primarily on the intradomiciliary application of residual insecticides [16]. For the current control program, third-generation synthetic pyrethroids, including beta-cyfluthrin (12.5% suspension concentrate [s.c.], at 25% active ingredient [a.i.]/m^2^), cyfluthrin (10% wettable powder [w.p.], at 50 mg a.i./m^2^), delatamethrin (10% s.c. or 5% w.p. at 25 mg a.i./m^2^), and lambda-cyhalothrin (10% w.p. at 30 mg a.i./m^2^) (GNMH communication), were used based on market availability [17]. The current policy for selecting villages to spray entails a 5% intradomiciliary prevalence threshold but relies on targeted surveys of presumed risk factors and suspected infestation [11], [15], namely “villages suspected of being infested with *R. prolixus* or *T. dimidiata*, where infestation was reported or in rural areas where the majority of the houses are constructed with mud walls and/or thatched roofs” [15]. However, if villages with low prevalences are visited unnecessarily, or villages with high prevalences are missed, such a policy may not necessarily maximize the effectiveness of limited resources. In a resource limited setting, developing a rational control program to sustain *T. dimidiata* village intradomiciliary prevalence below 5%, will depend upon ensuring that control efforts are targeted to villages with the highest risk of infestation.

From 2000–3, GNMH, the Japanese International Cooperation Agency (JICA), and the Universidad del Valle de Guatemala (UVG) with other collaborating instituions undertook a series of targeted and random surveys to assess *T. dimidiata* prevalance prior to vector control [1], [11], [15], [18]. This study makes use of the data gathered in the central department of Baja Verapaz and southeastern department of Jutiapa to compare the effectiveness of random and targeted surveys in determining villages at high risk for *T. dimidiata* infestation in these two regions. Specifically, our objective was to evaluate the capability of the random and targeted survey methods in directing control to villages at greatest risk of infestation by comparing the ability of environmental and/or domiciliary risk factors to predict intradomestic prevalence >5% by survey and department.

## Materials and Methods

### Datasets

**Figure 1:**
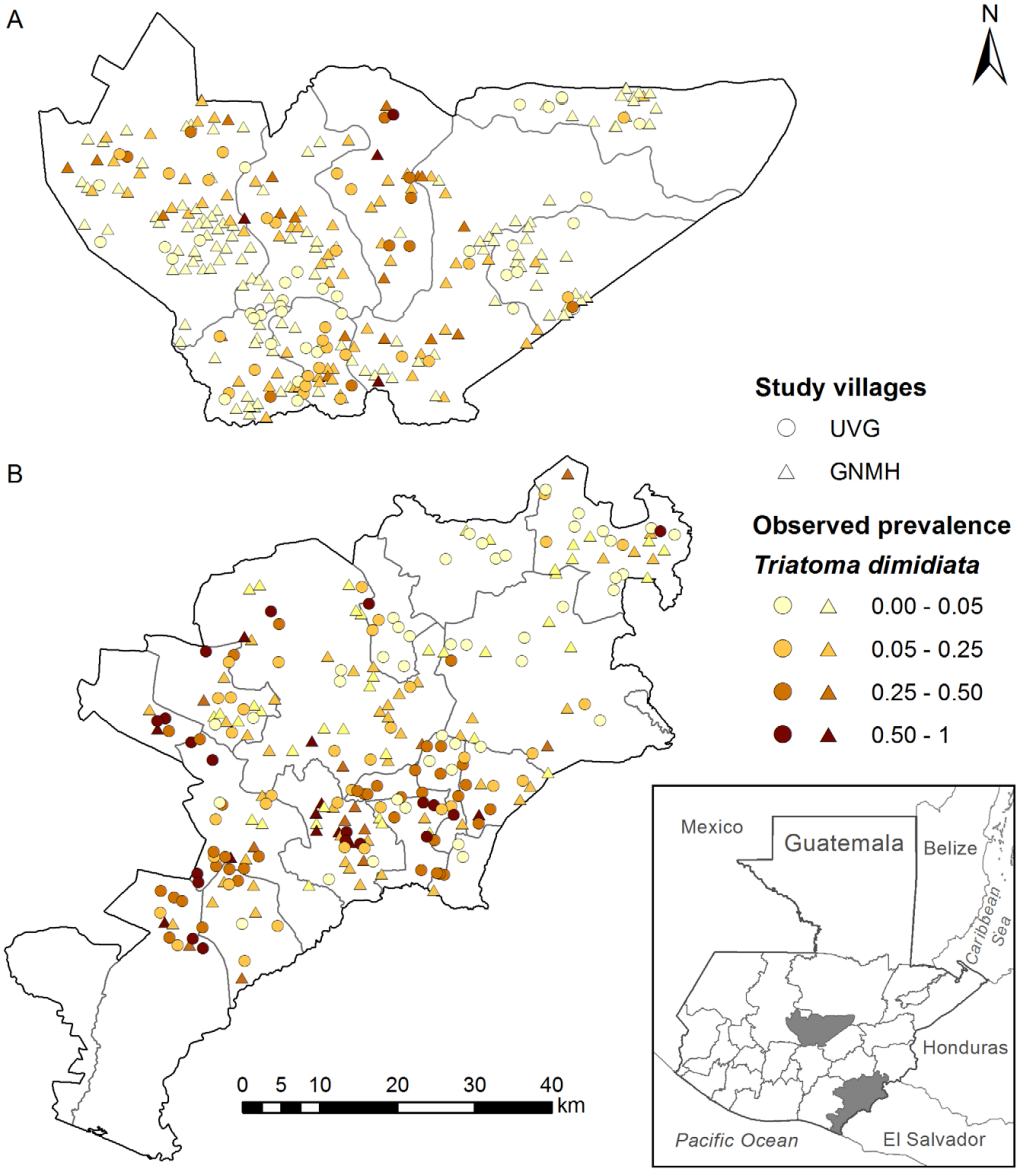
Map of the geographic distribution and intradomiciliary prevalences of villages analyzed. The location and intradomiciliary prevalences of villages analyzed in (A) Baja Verapaz and (B) Jutiapa. Each symbol represents a village, with circles symbolizing Universidad del Valle de Guatemala randomly sampled villages and triangles symbolizing Guatemala National Ministry of Health targeted villages. Shading indicates the level of intradomiciliary prevalence within each village. Inset: location of study departments within Guatemala and Central America. Note: Guatemala is divided into 22 departments and 331 municipalities [32] (www.state.gov/r/pa/ei/bgn/2045.htm). Health services, including vector control, are administered at the department level by each Health Area Authority [1].

*Triatoma dimidiata* intradomiciliary prevalence data at the village level for the departments^1^ of Baja Verapaz and Jutiapa from 2000–3 were obtained from randomized cross-sectional pre-spray surveys implemented by UVG [10] and from targeted cross-sectional pre-spray surveys performed by GNMH [11], [18]. These departments are positioned within two principal regions of *T. dimidiata* infestation. Baja Verapaz is located in the temperate and subtropical dry forests [19] of central Guatemala, 89.93°–90.81°W and 13.74°–14.56°N, encompassing an area of 2864 km^2^. Jutiapa is positioned in the subtropical moist forest [19] in the southeast, 89.50°–90.30°W and 13.74°–14.56°N, covering an area of 3318 km^2^. The geographic distribution of villages surveyed by department and study is illustrated in Figure 1. Here, department prevalence refers to the proportion of villages within each department that are intradomiciliary infested with *T. dimidiata*, and village prevalence refers to the proportion of infested domiciles within each surveyed village.

Specific details of data collection and survey design have been previously published [1], . In brief, both surveys analyzed here are subsets of larger studies aimed at determining triatomine prevalence in central and southeastern Guatemala prior to a vector control campaign. Baja Verapaz and Jutiapa were selected here due to similarities in the broad geographic coverage of sampled villages, the presence of significant *T. dimidiata* infestation with limited *R. prolixus* infestation [3], [4], [18], [20], and the locations of the departments in two different regions, central and southeastern Guatemala. Moreover, the departments were analyzed separately as the vector surveys were administered at the department level [1] and due to the location of the departments in two different Holdridge Life zones. Baja Verapaz occurs in the subtropical and warm temperate dry forest and Jutiapa occurs in the subtropical moist forest [21].

The random data set was derived from a cross-sectional survey supported by the Tropical Disease Research and Training program (TDR), World Health Organization no. 990545 and the Centers for Disease Control and Prevention CoAg U50 CCU021236 by UVG in collaboration with GNMH from 2000–3 [10]. In each municipality, villages and domiciles were selected randomly [10]. All eight municipalities in Baja Verapaz and 16 of 18 municipalities in Jutiapa were surveyed. Within these municipalities, georeferenced data was obtained from 79 villages and 1021 domiciles in Baja Verapaz and 162 villages and 2215 domiciles in Jutiapa. Entomological evaluation was conducted using an abbreviated man-hour collection method [3]. For each domicile selected, the intradomestic and surrounding peridomestic environments were surveyed manually for triatomines by two entomology technicians for 15–30 minutes, as determined subjectively by the size of the house [10].

The targeted data set was derived from cross-sectional entomological surveys carried out by GNMH in collaboration with JICA from 2000–3 [1], [11], [18]. Domiciles were selected from a sampling frame that excluded villages sampled in the random survey. Within Baja Verapaz, all eight municipalities were surveyed while 14 of 18 were examined in Jutiapa. In contrast to the random survey, study villages were targeted in rural areas on the basis of anecdotal surveys, suspected infestation, previous infestation, or presumed risk factors, e.g., domiciles with walls made of mud and/or roofs constructed of thatch [11], [15]. Georeferenced data were obtained from 262 villages and 5306 domiciles in Baja Verapaz and 244 villages and 2954 domiciles in Jutiapa. Entomological evaluation was also conducted by an abbreviated man-hour collection method [3]. The intradomestic and peridomestic environments of selected domiciles were searched manually for triatomines for 30 minutes by one entomology technician and for 15 minutes by two technicians [11]. These findings were later used by GNMH to target pyrethroid spraying to domiciles and peridomestic annexes in villages with intradomiciliary prevalences >5% [1], [10], [11], [15], [18].

Environmental and socioeconomic data were obtained from multiple sources and are described in Table 1. Covariate and georeferenced infestation data were imported into the GIS TNTmips 2008:74 (Microimages, Lincoln, NE). Layers were processed and linked geographically. With the exception of land cover, environmental covariate values were defined using the geographic coordinates for each village. For land cover, the proportion of each land cover class (forest, grassland, cropland, wetland, and settlement) within a 2 km buffer of each village was determined. Domiciliary construction data were then summarized by calculating the proportion of each domicile construction material per village. All data were then extracted by village and exported for statistical analysis. Data were displayed and mapped using ArcView GIS v. 9.2 (Environmental Systems Research Institute, Inc., Redlands, CA).

**Table 1:** Summary of environmental and socioeconomic databases used in analyses.

			Resolution			
Data type	Database	Source	Spatial	Temporal	Units	Citation
Environmental	Annual precipitation	WorldClim	1 km	1950–2000	mm	www.worldclim.org [33], [34]
	Digital elevation model	CGIAR-CSI	90 m	2004	m	www.csi-cgiar.org [35]
	LST daytime and nighttime mean, max, min	MODIS	1 km	2001–3	°C	lpdaac.usgs.gov [36]
	MIR mean, max, min	AVHRR/TFA	1 km	1992–6	°C	Hay 2006 [37]
	NDVI mean, max, min	MODIS	1 km	2001–3		lpdaac.usgs.gov [36]
	RH mean, max, min	CRU/UEA	10′	1961–90	%	www.cru.uea.ac.uk [38]
	Land cover	SERVIR	0.5 km	2005		www.servir.net [39]
Socioeconomic	House floor, wall and roof material	INE	Village	2002		www.ine.gob.gt/ [40]

Key to database abbreviations: LST, land surface temperature; MIR, middle infrared; NDVI, normalized difference vegetation index; RH, relative humidity; max, maximum average value; min, minimum average value. Key to database source abbreviations: CGIAR_CSI, Consultative Group for International Agriculture Research – Consortium for Spatial Information; MODIS, moderate resolution imaging spectroradiometer; AVHRR/TFA, advanced very high resolution radiometer transformed by temporal fourier analysis; CRU/UEA, Climate Research Unit,/University of East Anglia; INE, Instituto Nacional de Estadistica de Guatemala.

### Statistical analysis

Analysis of *T. dimidiata* pre-spray prevalence data was limited to those villages where at least five domiciles were surveyed. Similarities in the geographic distribution of villages between the two studies were maximized by excluding villages from one study when their distance to the closest village in the opposing study exceeded five kilometers. For the remaining villages, descriptive statistics of *T. dimidiata* village and department level prevalences were summarized by study and department.

Analyses of risk factors associated with *T. dimidiata* intradomiciliary prevalence at the village level for each department and study were carried out using univariate and multivariate logistic regression. First, univariate logistic regression models for grouped data were fitted to each of the grouped climatic variables (land surface temperature, normalized difference vegetation index, middle infrared reflectance, and relative humidity) to identify covariates in each category that best discriminated village prevalence. For ease of interpretation and direct comparison of climate characteristics between studies, variables were selected from the analyses of the UVG random data set in each department. Variables with a Wald's P>0.05 were excluded from further analyses due to the large number of significant covariates. The best fit model for each category was then selected on the basis of its Akaike weight (*w_i_*). Although the number of parameters for each model was the same in this investigation, the statistic provided a simple and easily interpretable measure for model comparison [22], [23].

The environmental and domiciliary risk factors associated with *T. dimidiata* village prevalence >5% was investigated by univariate logistic regression for each study by department. The outcome variable was defined by village as *T. dimidiata* intradomiciliary prevalence ≤ or >5%. Explanatory variables included climate variables selected from the discriminative univariate analyses, the remaining environmental covariates (elevation, precipitation, and land cover), and all domiciliary construction covariates. A logistic regression model was fitted to each covariate to define the odds of infestation associated with each potential risk factor.

Predictions of the probability of village prevalence >5% were then made by fitting a series of multivariate logistic regression models using a jackknife procedure, whereby a single village was excluded and an estimate of its predictive probability was made using the remaining data [24], [25]. This method maximizes the data used to estimate a villages predictive probability and allows for model validation using independent data [24]. All significant covariates from the logistic regression models were used to fit multivariate models. Predictive models of environmental and domiciliary covariates for each study by department were generated individually and together. Diagnostic statistics were generated to compare model accuracy. The area under the receiver-operator curve (AUC) was calculated to compare overall model performance and kappa (κ), sensitivity, specificity, positive predictive value (PPV), and negative predictive value (NPV) were calculated across the range of predicted probability thresholds. All statistical analyses were conducted in Stata/IC version 10 (Stata Corporation, College Station, TX, USA).

## Results

### Prevalence and geographic distribution

The geographic distribution and intradomiciliary prevalences of villages selected for analysis are shown by department and study in Figure 1. In Baja Verapaz, villages in all eight municipalities were incorporated into the analysis of both surveys and included 894 domiciles in 72 villages from the random study and 4403 domiciles in 212 villages from the targeted study, representing 16.86% and 49.65% of villages, respectively, and 66.51% of villages overall (n = 427). Village prevalence of *T. dimidiata* was highest in the northwest and southern regions of the department. Department prevalence was highest in the random survey at 51.4% (95% CI 39.9–62.9), but not significantly different from the targeted survey with a prevalence of 39.2% (95% CI 32.6–45.7). In contrast, village prevalence was significantly higher in the random survey (13.0%, 95% CI 10.9–15.2) than in the targeted survey (8.7%, 95% CI 7.8–9.5).

*T. dimidiata* was distributed throughout Jutiapa with village prevalences highest in the central and southern regions. In the random study, 1919 domiciles in 138 villages and 16 municipalities were used for analyses, while in the targeted study, 2243 domiciles in 108 villages and 14 municipalities were used for analyses, representing 17.95% and 14.04% of villages, respectively, and 31.99% of villages overall (n = 769). Again, department prevalence was not significantly different between the random (68.8%, 95% CI 61.1–76.6) and targeted (62.0%, 95% CI 52.9–71.2) surveys, but village prevalences were significantly higher in the random (22.7%, 95% CI 20.9–24.6) than in the targeted (6.9%, 95% CI 5.9–8.0) surveys.

### Environmental risk factors

The grouped climate variables that best explained *T. dimidiata* village prevalence are presented in Table 2. These covariates were used in all subsequent analyses.

**Table 2:** Significant grouped climate variables with highest Akaike weight (*w_i_*).

Department	Covariate	Coefficient (95% CI)	*P*	AICc^1^	*w_i_*
Baja Verapaz	LST daytime average (°C)	0.29 (0.21,0.37)	0.000	631.89	0.98
	NDVI minimum	−7.72 (−10.07,−5.38)	0.000	643.45	0.71
	MIR average (°C)	0.30 (0.22,0.38)	0.000	630.00	0.99
	RH minimum	−0.11 (−0.19,−0.02)	0.014	688.06	1.00
Jutiapa	LST daytime average (°C)	−0.38 (−0.46,−0.31)	0.000	1957.28	1.00
	NDVI average	8.05 (6.38,9.73)	0.000	1967.49	1.00
	MIR average (°C)	−0.44 (−0.51,−0.36)	0.000	1909.13	1.00
	RH maximum	0.21 (0.12,0.30)	0.000	2039.73	1.00

Key to covariate abbreviations: LST, land surface temperature; MIR, middle infrared; NDVI, normalized difference vegetation index; RH, relative humidity. Key to database statistical abbreviations: AICc: Akaike information criterion for small sample sizes; *w_i_*, Akaike weight.

Univariate logistic regression models were fitted to each of the grouped climate variables to determine the covariates that best discriminated intradomiciliary village prevalence. Model performance was evaluated by the selecting the covariate with the highest Akaike weight (*w_i_*).

Table 3 shows the significant results of the environmental risk factor analyses for village prevalence exceeding the 5% control threshold for each survey and department. For Baja Verapaz, the significant environmental risk factors were the same for both survey types and similarly describe the odds of infestation. The magnitude of the observed effect of each covariate with the exception of annual precipitation (equal impact) was greatest in the random study. An increase in the average daytime LST, average MIR, and proportion of cropland within a 2 km buffer of villages were associated with an increase in the risk of infestation. In contrast, minimum NDVI, minimum RH, and the proportion of evergreen forest within a 2 km buffer were associated with a decrease in the risk of infestation. Annual precipitation and elevation had weak negative effects.

**Table 3:** Estimates of effect of significant environmental risk factors on *Triatoma dimidiata* intradomiciliary prevalence >5%.

		Random survey		Targeted survey
Department	Risk factor	OR (95%CI)	*P*	OR (95% CI)	*P*
**Baja Verapaz**	Annual precipitation (mm)	**0.999** (0.997,0.9999)	0.040	**0.999** (0.998,0.9998)	0.011
	Elevation (m)	**0.996** (0.995,0.998)	0.000	**0.999** (0.998,0.9998)	0.015
	LST daytime average (°C)	**1.70** (1.34,2.16)	0.000	**1.24** (1.12,1.37)	0.000
	MIR average (°C)	**1.71** (1.33,2.20)	0.000	**1.20** (1.09,1.33)	0.000
	NDVI minimum	**7.34e-06** (1.40e-08,0.004)	0.000	**0.0005** (0.00002,0.02)	0.000
	RH minimum	**0.72** (0.57,0.92)	0.008	**0.85** (0.75,0.97)	0.013
	Cropland (%)	**292.52** (15.57,5496.13)	0.000	**10.66** (3.15,36.07)	0.000
	Evergreen forest (%)	**0.003** (0.0001,0.06)	0.000	**0.02** (0.004,0.16)	0.000
**Jutiapa**	Annual precipitation (mm)	**1.006** (1.004,1.008)	0.000	**1.003** (1.002,1.005)	0.000
	Elevation (m)	**1.003** (1.002,1.005)	0.001		
	LST daytime average (°C)	**0.57** (0.42,0.77)	0.000		
	MIR average (°C)	**0.40** (0.27,0.60)	0.000	**0.71** (0.54,0.94)	0.015
	NDVI average	**2.18e+5** (141.24,3.37e+08)	0.001	**8227.39** (4.33,1.56e+07)	0.019
	RH maximum	**1.70** (1.21,2.39)	0.002		
	Cropland (%)	**0.14** (0.02,0.81)	0.028		
	Grassland (%)	**12.36** (1.70,89.72)	0.013		
	Settlement (%)	**4.81e-07** (3.40e-11,0.007)	0.003		

Key to risk factor abbreviations: LST, land surface temperature; MIR, middle infrared; NDVI, normalized difference vegetation index; RH, relative humidity.

Univariate logistic regression models were developed to investigate the effect of each environmental covariate on *Triatoma dimidiata* intradomiciliary village prevalence >5% by survey and department. Odds ratios (OR) and 95% confidence intervals for significant risk factors are reported. Land cover classes represent the proportion of each land cover type within a 2 km buffer of analyzed villages.

In Jutiapa, fewer environmental risk factors were significant in the targeted study than in the random study. The direction of the relationships of similar significant risk factors in both studies was the same. As with the relationships of the covariates in the Baja Verapaz studies, the magnitude of the observed effects was greatest in the random study but not significantly different as the confidence intervals overlapped. For both studies, the average NDVI had a substantial positive effect on the risk of infestation, while the odds of infestation were negatively associated with the average MIR. In addition, the proportion of grassland with in a 2 km buffer of an infested village and the maximum RH were associated with an increased risk of infestation in the random study. Moreover, the average daytime temperature, and proportion of cropland and settlements within a 2 km buffer of infested villages were associated with a decreased risk of infestation.

### Domiciliary risk factors

Significant domicile construction risk factors associated with village prevalence >5% are shown in Table 4. Fewer villages contained data on domicile construction materials than environmental covariates in each study and department. In Baja Verapaz, 64 of 72 villages in the random survey and 160 of 212 villages in the targeted survey had corresponding construction data, while in Jutiapa 123 of 138 villages in the random survey and 89 of 108 villages in the targeted survey had data on domicile construction covariates. The effect of similar domicile construction materials in both departments was consistent among studies. Risk was higher in adobe walled domiciles and lower in aluminum roofed domiciles in Baja Verapaz. In Jutiapa, risk was higher in domiciles with dirt floors and roofs of aluminum or tile and lower in domiciles with floors made of clay tile or cement.

**Table 4:** Estimates of effect of significant domicile construction materials on *Triatoma dimidiata* intradomiciliary prevalence >5%.

			Random survey	Targeted survey
Department	Location	Risk factor	OR (95% CI)	*P*	OR (95% CI)	*P*
Baja Verapaz	Wall	Adobe	**5.76** (1.08–30.60)	0.004	**13.10** (3.38,50.58)	0.000
		Wood			**0.04** (0.004,0.46)	0.009
	Roof	Aluminum	**0.12** (0.01–0.94)	0.044	**0.11** (0.03,0.04)	0.001
		Tile			**8.04** (2.31,28.01)	0.001
Jutiapa	Floor	Cement slab			**0.09** (0.01,0.87)	0.037
		Cement tile	**0.05** (0.01–0.35)	0.003		
		Ceramic	**7.60e-11** (9.27e-18-0.001)	0.004		
		Clay tile	**3.66e-11** (1.25e-19-0.01)	0.016	**1.23e-12** (2.25e-24,0.67)	0.047
		Earth	**26.84** (5.64–127.79)	0.000	**8.81** (1.69,46.04)	0.010
	Wall	Brick	**0.001** (0.00001–0.37)	0.015		
		Block	**0.05** (0.004–0.56)	0.016		
		Stick & mud	**11.97** (1.04–137.44)	0.046		
		Palm & straw	**1.30e+16** (8.99–1.88e+31)	0.037		
	Roof	Aluminum	**7.64** (1.66–35.14)	0.009	**9.96** (1.63,60.80)	0.013
		Concrete	**1.90e-26** (2.45e-41-1.47e-11)	0.001		
		Tile	**0.15** (0.04–0.65)	0.011	**0.15** (0.03,0.79)	0.026

Univariate logistic regression models were developed to investigate the effect of each domicile construction material on *Triatoma dimidiata* intradomiciliary village prevalence >5% by survey and department. Odds ratios (OR) and 95% confidence intervals for significant risk factors are reported. Domicile construction risk factors represent the proportion of domiciles per village constructed with each material as determined by the 2002 national census of the Guatemalan National Institute of Statistics [40].

In both departments, village prevalence in each study was often associated with different risk factors. For example, the targeted survey in Baja Verapaz found an increased risk associated with tile roofed domiciles that was not detected in the random study. Moreover, the random survey in Jutiapa detected a series of associations with wall materials not observed in the targeted survey. In particular, walls constructed of stick and mud or palm and straw were associated with considerable increases in the risk of infestation. Brick and block walls had marked protective effects. Interestingly, the direction of the effect of similarly significant materials such as aluminum and tile roofs contrasted between departments.

### Predictive models

A summary of the performance of the multivariate logistic regression models ability to predict village prevalence >5% is presented in Table 5. Models were constructed using villages with data for both environmental and domicile construction covariates to allow for direct comparison. The area under receiver-operator curve (AUC) is the best measure of a model's overall discriminative ability [25], [26]. With the exception of the domicile construction material model in Baja Verapaz, the random models for both departments had reasonably good discriminative capacity and performed significantly better than the corresponding targeted models. All targeted models had poor discriminative capacity. Moreover, the environmental and combination models in the Baja Verapaz random surveys had similar predictive power and performed significantly better than the domicile construction material model. In the Jutiapa random surveys, no significant difference in predictive performance was detected between models.

**Table 5:** Diagnostic statistics for predictive models of *Triatoma dimidiata* intradomiciliary prevalence >5%.

		Accuracy measures					
Dept/Study	Model	AUC (95% CI)	Max κ	Sensitivity % (95% CI)	Specificity % (95% CI)	PPV % (95% CI)	NPV % (95% CI)
BV/UVG	ENV	0.84 (0.74,0.94)	0.56	76.5 (58.4,88.6)	80.0 (60.9,91.6)	81.3 (63.0,92.1)	75.0 (56.3,87.9)
	DOM	0.58 (0.44,0.72)	0.16	82.4 (64.8,92.6)	33.3 (17.9,52.9)	58.3 (43.3,72.1)	62.5 (35.9,83.7)
	ALL	0.84 (0.74,0.93)	0.51	64.7 (46.5,79.7)	86.7 (68.4,95.6)	84.6 (64.3,95.0)	68.4 (51.2,82.0)
BV/GNMH	ENV	0.65 (0.56,0.73)	0.24	80.3 (67.8,89.0)	46.5 (36.5,56.7)	48.0 (38.1,58.1)	79.3 (66.3,88.4)
	DOM	0.65 (0.56,0.74)	0.27	82.0 (69.6,90.2)	48.5 (38.4,58.7)	49.1 (39.5,59.6)	81.4 (68.7,89.9)
	ALL	0.65 (0.57,0.74)	0.19	68.9 (55.6,79.8)	59.6 (49.2,69.2)	51.2 (40.0,62.3)	75.6 (64.4,84.4)
JU/UVG	ENV	0.86 (0.78,0.93)	0.57	82.9 (72.7,90.0)	75.6 (59.4,87.1)	87.2 (77.2,93.4)	68.9 (53.2,81.4)
	DOM	0.77 (0.68,0.87)	0.51	91.5 (82.7,96.2)	56.1 (39.9,71.2)	80.7 (70.9,87.8)	76.7 (57.3,89.4)
	ALL	0.84 (0.76,0.92)	0.57	79.3 (68.6,87.1)	80.5 (64.6,90.6)	89.4 (79.2,94.8)	66.0 (51.1,78.4)
JU/GNMH	ENV	0.67 (0.55,0.78)	0.35	64.7 (50.0,77.2)	71.1 (53.9,84.0)	75.0 (59.4,86.3)	60.0 (44.4,73.9)
	DOM	0.65 (0.53,0.77)	0.30	66.7 (52.0,78.9)	63.2 (46.0,77.7)	70.8 (55.7,82.6)	58.5 (42.2,73.3)
	ALL	0.64 (0.52,0.76)	0.30	54.9 (40.5,68.6)	57.9 (40.9,73.3)	63.6 (47.7,77.2)	48.9 (33.9,64.0)

Key to department and study abbreviations: Dept, department; BV, Baja Verapaz; JU, Jutiapa; UVG, Universidad del Valle de Guatemala; GNMH; Guatemala National Ministry of Health. Key to model abbreviations: ENV, environmental model; DOM, domicile construction material model; ALL, combination of census and environmental models. Key to accuracy measure abbreviations: AUC, area under receiver-operator curve; Max κ, maximum kappa; PPV, positive predictive value; NPV, negative predictive value.

Multivariate logistic regression models were developed to estimate the predictive probability of *Triatoma dimidiata* intradomiciliary village prevalence >5%. For each department and study, predictive models of environmental and domicile construction risk factors were developed separately and together. Overall model accuracy was compared using the area under the receiver-operator curve (AUC). Sensitivity, specificity, positive predictive value (PPV), and negative predictive value (NPV) were calculated using the probability threshold with maximum value of kappa (κ).

κ, sensitivity, specificity, PPV, and NPV all vary with the selection of the predicted probability threshold. The maximum κ obtained for each model is reported in Table 5 and the remaining accuracy measures are calculated using the corresponding threshold. All models from random surveys, with the exception of the domicile construction material model in Baja Verapaz, performed significantly better than chance alone. With regard to the random surveys, predictions based on environmental covariates had the greatest accuracy in Baja Verapaz, and environmental and combination models had similar and greater accuracy than domicile construction covariates in Jutiapa.

## Discussion

Sustained control of *T. dimidiata* depends on the accurate identification of areas at greatest risk of infestation in order to efficiently target limited resources. In their efforts to eliminate Chagas disease from Guatemala, vector control initiatives have relied on targeted surveys of villages with presumed risk factors or suspected infestation [11], [15], [16], however, their performance has not been evaluated. The data sets analyzed here afforded a unique opportunity to compare the abilities of random and targeted baseline cross-sectional surveys of *T. dimidiata* village prevalence conducted concurrently in time and space and resulted in several important findings relevant to *T. dimidiata* vector control: 1) random surveys performed just as well if not better than targeted surveys at defining the risk of *T. dimidiata* infestation, 2) intradomiciliary and environmental risk factor associations with *T. dimidiata* prevalence >5% varied with geographic location, 3) environmental risk factors provide additional insight into the intradomiciliary risk of *T. dimidiata* prevalence exceeding the control threshold, and 4) predictive modeling has a role to play in directing *T. dimidiata* control in Guatemala if data sets are appropriately defined and expectations realistic. To our knowledge, this is the first study to compare targeted and random surveys for *T. dimidiata* and has implications for *T. dimidiata* control in Guatemala and Central America.

The failure of the targeted surveys to detect higher department and village prevalences than random surveys was surprising. These findings illustrate that the methods used to focus targeted surveys were not any better than random sampling at determining villages at greatest risk for *T. dimidiata* infestation. Therefore, presuming risk factors and infestation was inadequate and when initiating a program, efforts should favor risk factor evaluation and validation prior to targeting surveys or favor random sampling, as the results could reflect insufficiently defined risk factors and/or the assumption of geographic similarity in risk factor effect. Although, the findings could also be attributed to greater experience and expertise among UVG surveyors who conducted the random surveys [10], [18].

The analysis of the intradomiciliary and environmental risk factors further supports the notion that the poor performance of the targeted surveys resulted at least in part from insufficiently defined risk factors and geographic heterogeneity in their effect. The limited ability of the presumed risk factors is illustrated by our ability to detect further robust relationships with additional indicators in the analysis of the targeted survey data. Moreover, many of the risk factors contrasted in their significance and the direction of their effect between departments. Even the presumed risk factors contrasted in their significance between departments. Walls of adobe had strong positive association with *T. dimidiata* village prevalence exceeding the control threshold in Baja Verapaz only, while walls of stick and mud were significant in Jutiapa only. The lack of a significant association with thatch roofs in both surveys and departments likely reflects the inclusion of this risk factor to aide in the targeting of *R. prolixus* [2].

Particularly interesting was the contrasting relationship between tile roofs and infestation exceeding the control threshold in the departments. Tile roofs had a protective effect in Jutiapa but were associated with increased risk in infestation in Baja Verapaz. A similar increased risk was detected in Costa Rica where it was suggested that the presence of spare roofing tiles in the peridomestic environment provided suitable habitat for *T. dimidiata* [27]. Peridomestic surveys associated with the targeted study in Baja Verapaz found established *T. dimidiata* populations, although specific peridomestic environments were not reported [18]. These findings suggest the potential for roofing tiles to play a similar role in Baja Verapaz. Peridomestic populations are also present in Jutiapa [10] but were not reported here. The protective effect could indicate tile roofs in this region are associated with improved living conditions, thus, limiting intradomestic populations. In addition, previous studies in Jutiapa found no direct association between intradomestic and peridomestic infestation [10], indicating that spare roofing tiles in the peridomestic environment may be of little significance to intradomestic *T. dimidiata* populations in Jutiapa. More detailed studies are needed to clarify the variation of risk factors in different ecological settings.

Moreover, the analysis of the environmental covariates also illustrated the geographic heterogeneity in risk factor association with *T. dimidiata* infestation >5% and indicated their potential value as indicators of infestation exceeding the control threshold. For example, villages with higher temperatures, increasingly barren landscapes, and more cropland were associated with increases in prevalence above the threshold in Baja Verapaz, while in Jutiapa an increase in vegetated landscapes, the proportion of grassland, and maximum RH were associated with increased risk of infestation. Future surveys should evaluate the inclusion of environmental risk factors as an aide in focusing control efforts. Furthermore, the observed geographic heterogeneity of both domiciliary and environmental risk factors illustrates the need to evaluate risk factors prior to use in a particular geographic location and the risk in extrapolating findings beyond the geographic limits for which they were defined. This observed heterogeneity is even more important in light of recent molecular studies suggesting that *T. dimidiata* in Guatemala represents a geographically diverse species complex [28], [29] with one study elevating a member to specific status [28].

The findings from the predictive models indicate the potential for this type of analysis and risk mapping to aide in directing *T. dimidiata* control to regions at greatest risk as well as support the findings discussed above with regard to the abilities of the random surveys and potential value of environmental covariates. The reasonably high sensitivities and PPV's among the best performing models from the random surveys in both departments indicate marginal resource loss when applying control measures. Similarly, the respectable specificity and NPV's suggest that the number of positive villages missed would be moderately low. Moreover, the performance of the targeted surveys suggest that they might have a limited role to play in generating predictive models if risk factors are adequately defined first and sensitivity and PPV are reasonably good in targeting high risk villages. Although, one would have to accept a significant number of positive villages would be excluded from control due to the expected low specificity and NPV.

Also notable among the results were the performance of the environmental covariates in predicting risk of *T. dimidiata* prevalence >5%. The predictive performance of environmental models was just as good if not significantly better than domicile construction material models. As mentioned previously, this could relate to insufficiently defined risk factors and/or geographic heterogeneity in their effect. In addition, it could be that the association with environmental covariates is related to the peridomestic populations in these regions, implying that peridomestic populations give rise to intradomestic populations or are in constant movement from one environment to the other. However, it might also be that the environmental conditions that are present in a region dictate the domicile construction materials used and represent confounding relationships with existing covariates and subsequently the type of construction defines the temperature and relative humidity inside the domicile. This could explain why the predictive models combining both environmental and domicile construction risk factors failed to improve overall model performance. Future models might be improved by the inclusion of intradomiciliary physical variables such as temperature and relative humidity.

As with any study, it is important to point out the limitations that exist. First, the targeted sample is biased by the exclusion of villages sampled in the random survey. Differences in our results could reflect differences in the villages sampled, although, we tried to account for significant variation by comparing geographically similar villages. Secondly, the findings are relevant to surveys conducted by the man-hour collection method, which is labor intensive with small reward and likely varies with expertise and experience [30]. Other collection methods could be less biased by experience, more consistent and efficient, and better able to define risk factors. Thus, the lack of the results could reflect variation in the ability to adequately detect bugs and not the absence of bugs and their associations with the risk factors. In addition, neither study was designed with our analysis in mind and therefore doesn't allow for optimal comparison. Future studies could control for this by selecting villages from the same sample frame, choosing the same number of domiciles in each village to survey, and conducting surveys with similarly experienced technicians. In addition, a true comparison of survey effectiveness should balance scientific abilities against their cost, with decisions made accordingly.

Nonetheless, the findings from our study lead us to several recommendations for *T. dimidiata* control in Guatemala and Central America. First, a priori knowledge, a prerequisite for targeted surveys, was not reliable for *T. dimidiata* surveys in Guatemala. Random surveys performed just as well if not better than targeted surveys, and have the additional benefit of risk factor detection, resulting from increased sample heterogeneity. Therefore, random surveys should be considered over targeted surveys if the reliability of the risk factors used to target surveys has not been evaluated. Secondly, risk factors for *T. dimidiata* infestation should be characterized for a particular geographic location through proper epidemiological investigation. One should keep in mind that the risk of extrapolation error increases as the distance from the source from which it was defined increases [31]. Furthermore, the role of environmental risk factors should be considered in addition to traditional intradomiciliary construction risk factors when investigating the risk of *T. dimidiata* infestation. Finally, our results indicate that predictive modeling has a role to play in targeting *T. dimidiata* control as long as the surveillance data is appropriately defined and/or model error is acceptable. It should be stressed that random surveys are not simply a luxury but an investment in programs. Future surveys should weigh their benefits as well cost when initiating a vector control program.

In conclusion, sustained control of *T. dimidiata* will depend on accurate and thorough epidemiological investigation. It is essential that the sample surveys on which decision making is based are evaluated to ensure that policy is not formed blindly and resources are not wasted. Here we show that a priori knowledge was not reliable in defining *T. dimidiata* risk in Guatemala. The random survey performed just as well if not better than the targeted survey. Moreover, our findings illustrate the blanket application of “presumed risk factors” should be applied with caution and based on initial scientific evaluation to ensure geographic extrapolation is appropriate. Future targeting of *T. dimidiata* surveys should also include environmental risk factors as they performed just as well if not better than domicile construction covariates at detecting infestation exceeding the control threshold. Random surveys were generally more successful at detecting risk factors and predicting infestation than targeted surveys and should be applied over targeted surveys when risk factor identification, predictive modeling and extrapolation to the general populations is the goal. These findings illustrate the need for studies that are well defined, geographically specific, and based on reliable epidemiological investigation.
